# Molecular landscape of IDH-mutant astrocytoma and oligodendroglioma grade 2 indicate tumor purity as an underlying genomic factor

**DOI:** 10.1186/s10020-022-00454-z

**Published:** 2022-03-14

**Authors:** Binghao Zhao, Yu Xia, Fengchun Yang, Yaning Wang, Yuekun Wang, Yadong Wang, Congxin Dai, Yu Wang, Wenbin Ma

**Affiliations:** 1 Departments of Neurosurgery, Peking Union Medical College Hospital, Chinese Academy of Medical Sciences and Peking Union Medical College, Beijing, 100730 People’s Republic of China; 2 Department of Neuropathology Peking Union Medical College Hospital, Chinese Academy of Medical Sciences and Peking Union Medical College, Beijing, People’s Republic of China; 3 State Key Laboratory of Complex Severe and Rare Diseases, Peking Union Medical College Hospital, Chinese Academy of Medical Science and Peking Union Medical College, Beijing, People’s Republic of China; 4 Institute of Medical Information, Chinese Academy of Medical Sciences and Peking Union Medical College, Beijing, People’s Republic of China; 5 Department of Thoracic Surgery, Peking Union Medical College Hospital, Peking Union Medical College and Chinese Academy of Medical Sciences, Beijing, People’s Republic of China; 6 Department of Neurosurgery, Beijing Tongren Hospital, Capital Medical University, Beijing, People’s Republic of China; 7 China Pituitary Disease Registry Center, Chinese Pituitary Adenoma Cooperative Group, Beijing, People’s Republic of China; 8 China Alliance of Rare Diseases, Beijing, People’s Republic of China

**Keywords:** IDH-mutant, Astrocytoma, Oligodendroglioma, Tumor microenvironment, Tumor purity

## Abstract

**Background:**

IDH-mutant astrocytoma and oligodendroglioma have an indolent natural history and are recognized as distinct entities of neoplasms. There is little knowledge on the molecular differences between IDH-mutant astrocytoma and oligodendroglioma grade 2. Therefore, we investigated the multiomics and clinical data regarding these two types of tumors.

**Method:**

In silico analyses were performed around mRNA, somatic mutations, copy number alternations (CNAs), DNA methylation, microRNA (miRNA), epigenetics, immune microenvironment characterization and clinical features of the two types of gliomas. A diagnostic model incorporating tumor purity was further established using machine learning algorithms, and the predictive value was evaluated by receiver operative characteristic curves.

**Results:**

Both types of gliomas shared chromosomal instability, and astrocytomas exhibited increased total CNAs compared to oligodendrogliomas. Oligodendrogliomas displayed distinct chromosome 4 (chr 4) loss, and subtyping of chr 7 gain/chr 4 loss (+ 7/− 4) presented the worst survival (P = 0.004) and progression-free interval (PFI) (P < 0.001). In DNA damage signatures, oligodendroglioma had a higher subclonal genome fraction (P < 0.001) and tumor purity (P = 0.001), and astrocytoma had a higher aneuploidy score (P < 0.001). Furthermore, astrocytomas exhibited inflamed immune cell infiltration, activated T cells and a potential response to immune checkpoint inhibitors (ICIs), while oligodendrogliomas were more homogeneous with increased tumor purity and decreased aggression. The tumor purity-involved diagnostic model exhibited great accuracy in identifying astrocytoma and oligodendroglioma.

**Conclusion:**

This study addresses the similarities and differences between IDH-mutant astrocytoma and oligodendroglioma grade 2 and facilitates a deeper understanding of their molecular features, immune microenvironment, tumor purity and prognosis. The diagnostic tool developed using machine learning may offer support for clinical decisions.

**Supplementary Information:**

The online version contains supplementary material available at 10.1186/s10020-022-00454-z.

## Introduction

Incorporating the molecular landscape and molecular alternations into brain tumor classification and grading is driving continuing revolution in the field of neuro-oncology (Louis et al. 2016). In diffusely infiltrating gliomas, the mutational status of isocitrate dehydrogenase (IDH) and other molecular features determines biological behavior and defines the diagnostic category of IDH-mutant (IDH-mt) astrocytoma and 1p/19q codeletion oligodendroglioma (Louis et al. 2016). The recent World Health Organization (WHO) Classification of Tumors of the Central Nervous System (CNS) recommends stratification of IDH-mt glioma into WHO grade 2–4 for astrocytoma and grade 2–3 for oligodendroglioma based on their neuropathological features (Louis et al. 2021). Several studies have demonstrated that specific genetic alterations preclude the use of histology for predicting glioma outcomes. The presence of *CDKN2A/B* homozygous deletion is associated with decreased survival in IDH-mt glioma grades 2 and 3 (Cimino and Holland 2019; Cimino et al. 2017). *RB1* homozygous deletion, *PIK3CA* pathogenic mutations, *PDGFRA* amplification, and *MYCN* amplification have also been linked to worse survival (Aoki et al. 2018; Shirahata et al. 2018). Additionally, shorter survival was suggested in a series of hypermutated, mismatch repair-deficient IDH-mt gliomas (Touat et al. 2020).

The tumor microenvironment (TME) is boosted as complex milieu consisting of factors regulating tumor growth, as well as nutrients, chemokines and other non-cancerous cell types such as immune cells, fibroblasts, endothelial cells and normal epithelial cells. Tumor purity represents the proportion of tumor cells (0–100%) in the admixture ever estimated by the pathologists via visual or image analysis. However, it can be inferred with new computation methods. Heterogeneity of tumor cells is regarded as another surrogate feature of diffuse gliomas and a potential cause of treatment failure. Single-cell sequencing studies have highlighted transcriptional heterogeneity in regulatory programs covering the cell cycle and cellular states (Neftel et al. 2019; Venteicher et al. xxxx), nevertheless, bulk sequencing has indicated obvious heterogeneity in somatic drivers, such as *EGFR* and *PDGFRA*, as well as in tumor mutation burden (TMB) (Ceccarelli et al. 2016; Snuderl et al. 2011; Suzuki et al. 2015; Szerlip et al. 2012). Specifically, there is also genetic and epigenetic heterogeneity in IDH-mt astrocytoma and oligodendroglioma grade 2, however, the new version of the WHO CNS tumor classification does not provide updated detailed molecular variance reflecting clinical behaviors regarding such tumors. Additionally, further classification of these two entities remains poor, and improving our understanding of these entities would facilitate biological behavior understanding, neuropathology diagnosis and treatments. We devised this study based on multiomics data investigating the similarities and heterogeneity between the two entities to offer further knowledge on their tumor microenvironment (TME), malignancy, treatment and diagnosis.

## Methods and materials

### Data collection and preprocessing

Multiomics data for IDH-mt glioma grade 2, that is, mRNA expression, somatic mutation, CNA, DNA methylation, microRNA (miRNA), epigenetics data (reversed-phase protein arrays (RPPA) to explore protein expression and patient demographic information, were accessed from The Cancer Genome Atlas (TCGA) (*RNA-Seq Cohort*) and the Chinese Glioma Genome Atlas (CGGA) (*RNA-Seq Cohort*). These data were processed as described previously (Chen et al. 2019; Zhang et al. 2020). Details regarding the datasets are provided in Additional file 9: Table S1. RNA sequencing data in FPKM format were directly downloaded from the Genomic Data Commons Datga Portal (GDC) (https://portal.gdc.cancer.gov/) and CGGA repositories (http://www.cgga.org.cn/) and converted into transcripts per kilobase million (TPM) format (Wagner et al. 2012). All mRNA expression data underwent log2 (TPM + 1) transformation. Microsatellite instability (MSI) is always caused by epigenetic disorders or alterations in DNA mismatch repair (dMMR) markers. The assigned MSI data for evaluating tumor samples were calculated using the MSI monodinucleotide assay or hg38 sequencing per recommendations. Use of all of these samples was approved by the ethics committee in each repository.

Most importantly, samples were filtered to include grade 2 cases, IDH mutations only (*IDH1* or *IDH2*), astrocytoma and oligodendroglioma. Following the fifth WHO Classification on CNS Tumors and The Consortium to Inform Molecular and Practical Approaches to CNS Tumor Taxonomy (cIMPACT-NOW) updates, IDH-mt astrocytomas with homozygous deletion of *CDKN2A/B* were removed in particular (Louis et al. 2021). For the special type of oligoastrocytoma, the IDH-mt incorporating 1p/19q codeletion tumors were assigned to oligodendroglioma, IDH-mt only tumors were assigned to astrocytoma, and the others were removed (Louis et al. 2021). Neuropathological diagnosis was made prior to histological diagnosis, that is, if the two diagnoses were contradictory, we considered the neuropathological results as the diagnosis (Louis et al. 2021).

### Somatic mutation and copy number alternations analyses

We defined nonsynonymous mutations incorporating frameshift mutations, inframe mutations, missense mutations, nonsense mutations, and splice site mutations reflecting somatic mutations and recognized them as components of the TMB. For CNA analyses, we applied GISTIC_2.0 to identify significantly amplified or deleted genomes. The specific burden of copy number loss or gain was calculated as the total number of genes with copy number alternations at the focal and arm levels (Mermel et al. 2011). SubMap and GISTIC_2.0 software were used and are freely accessed on GenePattern (https://cloud.genepattern.org).

### Biological functional analysis

We applied the “clusterProfiler” package for Gene Ontology (GO) and Kyoto Encyclopedia of Genes and Genomes (KEGG) pathway functional analyses (Yu et al. 2012). Gene set enrichment analysis (GSEA) and gene set variation analysis (GSVA) were conducted to enrich hallmark gene sets obtained from the Molecular Signatures Database (MSigDB) (v7.1) (Subramanian et al. 2005). Input gene panels were ranked in descending order according to their log2-fold change (FC) values. A Benjamini–Hochberg adjusted P-value < 0.05 was considered significant.

### Evaluation on DNA damage repair signature and tumor purity

Cancer subtypes are often characterized by tumor-specific patterns of chromosomal arm-level alterations, including lung, esophageal, and bladder tumors (Hoadley et al. 2014). Oligodendroglioma with special chromosome arm-level alternations of 1p/19q codeletion was characterized to reveal its responsiveness to chemoradiotherapy regimens (Cairncross et al. 2013). Glioma tissues contain abundant associated nontumor cells within their microenvironment, which are represented by stromal and immune cells. We are deeply aware that even though nontumor cells dilute the tumor purity, function of tumor and nontumor cells subtly ensures homeostasis for gliomagenesis, malignancies, progression, treatment resistance and other diverse pathological roles (Zhang et al. 2017). To date, there is scarce knowledge regarding the characteristics of the DNA damage repair (DDR) and tumor cells regarding the various purities of these two entities. Samples from TCGA dataset were analyzed, and the CNA was determined from an Affymetrix SNP 6.0 array. We adopted the ABSOLUTE algorithm as an established pipeline to generate segmented absolute copy numbers and quantify tumor sample purity and other DDR-related molecular features with the distinct scores (Carter et al. 2012). Detailed data and methods can be found in a previous study (Taylor et al. 2018).

### Glioma cluster profiling

Previous studies have identified different clusters from TCGA data that contain RNA-seq, methylation, miRNA, copy number data, etc. for gliomas (Brat et al. 2015; Ceccarelli et al. 2016). The TCGA investigator study defines R1–R4 (RNA-seq clusters), M1-M5 (methylation clusters), mi1-mi4 (miRNA clusters) and C1–C3 (CNA clusters) subcategories (Brat et al. 2015),the Ceccarelli study defines LGr1-4 (panglioma RNA-seq clusters) and LGm1-6 (panglioma DNA methylation) subcategories (Ceccarelli et al. 2016). Differentially expressed multiomics data between astrocytoma and oligodendroglioma were processed and used.

### Quantifying the immune microenvironment

To quantify the immune and stromal cell infiltration patterns, we applied the following robust and highly informative methods: TIMER (6 cell types), CIBERSORT (22 cell types) (Newman et al. 2015), CIBERSORT-ABS (22 cell types), quanTIseq (11 cell types) (Finotello et al. 2019), MCPcounter (11 cell types) (Becht et al. 2016), Xcell (39 cell types) (Aran et al. 2017) and EPIC (8 cell types) (Racle et al. xxxx). Differentially expressed immune cells between the two entities are presented. Using the ESTIMATE algorithm, we calculated the immune and stromal scores to predict the cellular infiltration level (Yoshihara et al. 2013). Immune microenvironment signatures and biomarkers were obtained from the literature, and the relative abundance of these signatures was quantified using single-cell gene set enrichment analysis (ssGSEA) with the IOBR algorithm (Zeng et al. 2547). The potential response to checkpoint immunotherapy was evaluated using the immune phenotype (IPS) algorithm obtained in The Cancer Immunome Atlas (TCIA, https://tcia.at/) and the TIDE score captured in Tumor Immune Dysfunction and Exclusion (TIDE, http://tide.dfci.harvard.edu/) [ (Charoentong et al. 2017; Jiang et al. 2018)]. A higher IPS score and lower TIDE score yields a favorable immunotherapeutic response, as described previously (Charoentong et al. 2017; Jiang et al. 2018).

The gene signatures for exhausted CD8 + T cells (GET), immune cytolytic activity (CYT) or T cell inflamed gene expression profile (GEP) were obtained from previous studies (Ayers et al. 2017; Rooney et al. 2015; Zhao et al. 2021). Briefly, the CYT signature included *GZMA* and *PRF1*, and the GEP signature was composed of *CCL5*, *CD27*, *CD274*, *CD276*, *CD8A*, *CMKLR1*, *CXCL9*, *CXCR6*, *HLA-DQA1*, *HLA-DRB1*, *HLA-E*, *IDO1*, *LAG3*, *NKG7*, *PDCD1LG2*, *PSMB10*, *STAT1*, and *TIGIT*. The relative levels were quantified by ssGSEA score.

### Machine learning pipeline for astrocytoma-oligodendroglioma gene panel identification

First, we applied the random forest (RF) algorithm to select the prognosis-related differentially expressed genes (DEGs) between astrocytoma and oligodendroglioma using |logFC|> 1 and false discovery rate (FDR) < 0.05 criteria, and these DEGs were defined as the astrocytoma-oligodendroglioma gene panel (A-O panel). Next, we used adaptive boosting (AdaBoost), gradient boosting decision tree (GBDT), and extreme gradient boosting (XGBoost) algorithms through “RandomForest”, “adabag”, “gbm” “xgboost” packages to establish models to identify astrocytoma and oligodendroglioma. These tree-boosting pipelines are highly scalable end-to-end tree boosting systems with justified weighted quantile sketch for efficient proposal calculation, besides, could be regarded as novel sparsity-aware algorithms for parallel tree learning and effective cache-aware block structures for out-of-core tree learning. Over other machine learning methods, they can give efficient and state-of-the-art results on many standard classification benchmarks. Samples were randomly split into a training set (n = 151 in TCGA) and a test set (n = 65) at a ratio of 7:3 in seed set of 1, 2, 3, 4. In the training set, we gave the relative importance and discrimination power of every factor in the A-O panel and tumor purity with the best predictive value. The predictive accuracy was determined by minimizing the error rate and maximizing the area under the receiver operative curve (ROC). The results were reported from the test set, because train sets had the accuracy of 1 in these algorithms. ROCs with area under the curve (AUC) were plotted to assess performance metrics. Specificity, sensitivity, positive predictive value, negative predictive values were manually calculated. Six critical genes from A-O panel of great importance together shared by algorithms were selected, the predictive/diagnostic model was quantified by “A-O Panel Classifier” with multivariable cox regression analysis on critical genes. That was formula: A–O Panel Classifier = $\sum_{i=1}^{n} \beta ixi$, where *β*_*i*_ is the coefficient and *x*_*i*_ is the z-score–transformed relative expression value of each important gene.

### Statistical analysis

Spearman’s or Pearson’s correlation coefficients were estimated and tested. For normally distributed continuous data, we used Pearson’s test; for not normally distributed continuous data and ranked ordinal data, we used Spearman’s test. Specified methods were addressed. For comparisons between two groups, the variables were analyzed by Wilcoxon rank-sum test (the Mann–Whitney U test); for comparisons between more than two groups, the Kruskal–Wallis (K–W) test was used. Fisher’s exact test was used to detect statistical association between categorical variables. The Kaplan–Meier method was used to estimate survival curves, strategy was implemented to produce survival curves, and the log-rank (Mantel-Cox) test was used to compare survival distributions. We applied a univariable Cox proportional hazard regression model to calculate the hazard ratios (HRs) and a multivariable Cox model to determine independent prognostic factors. All statistical analyses were performed using R software (version 3.5.3), and two-sided P-values < 0.05 were considered statistically significant. R packages used are specified in different parts of the manuscript.

## Results

### The somatic mutation landscape

**Fig. 1:**
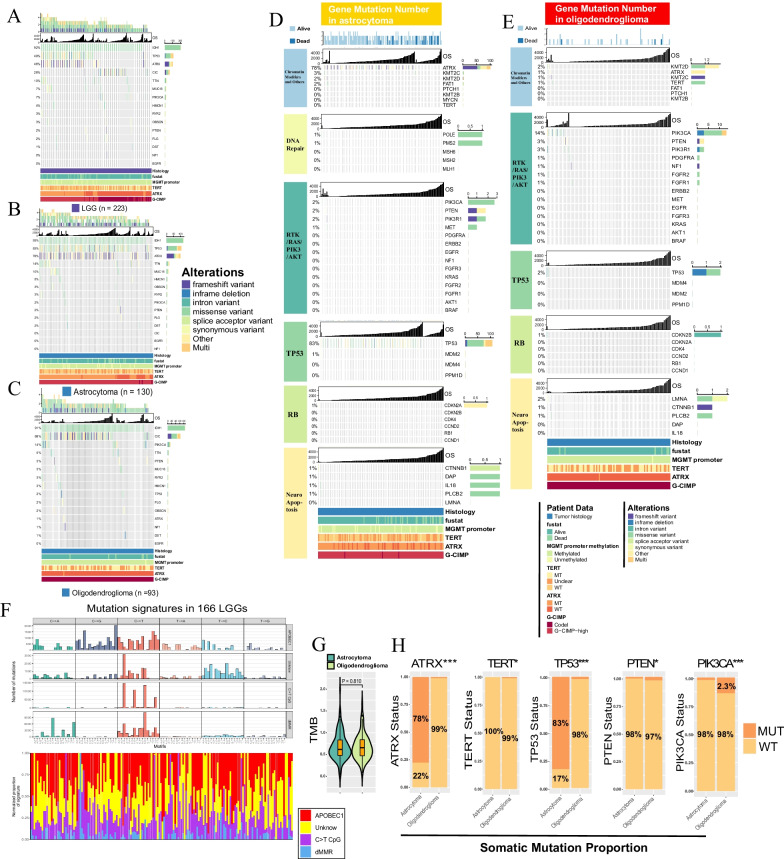
Somatic mutation landscape of IDH-mt astrocytoma and grade 2oligodendroglioma. **A** Somatic mutations in astrocytoma + oligodendroglioma (n = 223). **B** Somatic mutations in astrocytoma (n = 130). **C** Somatic mutations in oligodendroglioma (n = 93). **D** Somatic mutation frequency of genes in key pathways of astrocytoma. **E** Somatic mutation frequency of genes in key pathways of oligodendroglioma. Each column represents an individual patient. The number on the left indicates the mutation frequency, with a bar plot showing the proportion of each variant type. Gene names are indicated on the right. **F** COSMIC mutation signatures in astrocytoma + oligodendroglioma (n = 166) by APOBEC1, C > T CpG, dMMR and unknown groups. **G**. TMB value between astrocytoma and oligodendroglioma (Wilcoxon p = 0.81). **H** Proportion of mutated and wild type *ATRX*, *TERT*, *TP53*, *PTEN*, and *PIK3CA* genes between astrocytoma and oligodendroglioma (*Chi-square test p < 0.05; ***p < 0.001). *TMB* tumor mutation burden

The work flow of this study is shown in Additional file 1: Fig. S1. In total, 235 IDH-mt, grade 2 patients (140 astrocytoma, 95 oligodendroglioma) in TCGA and 211 patients (116 astrocytoma, 95 oligodendroglioma) in CGGA were included. Using TCGA somatic mutation data, astrocytoma plus oligodendroglioma (defined as low-grade glioma (LGG)) exhibited the most frequent somatic mutations in *IDH1* (92%), followed by *TP53* (49%), *ATRX* (46%), and *CIC* (28%). The top four mutated genes were *IDH1* (93%), *TP53* (83%), *ATRX* (78%), and *TTN* (14%) in astrocytoma and *IDH1* (93%), *CIC* (68%), *PIK3CA* (14%), and *TTN* (5%) in oligodendroglioma (Fig. 1A–C). Astrocytoma had a 78% mutated frequency for *ATRX* in chromatin modifiers and other pathways and an 83% mutated frequency for *TP53* in the *TP53* pathway; oligodendroglioma displayed a 14% mutated frequency for *PIK3CA* in the RTK/RAS/PIK3/AKT pathway (Fig. 1D–E). Using mutation data in the COSMIC database (https://cancer.sanger.ac.uk/cosmic), 166 LGGs were divided into APOBEC1, C  T CpG, dMMR and unknown groups (a lack of notable characteristics) using nonnegative matrix factorization (NMF). Interestingly, C  T was prominent throughout the four groups (Fig. 1F). There was no significant difference in TMB between the two tumor entities; astrocytoma exhibited higher mutation rates of *ATRX* and *TP53*, and oligodendroglioma presented higher mutated rates of *TERT*, *PTEN* and *PIK3CA* (Fig. 1G, H).

### Copy number alternations and related signatures between the two entities

**Fig. 2:**
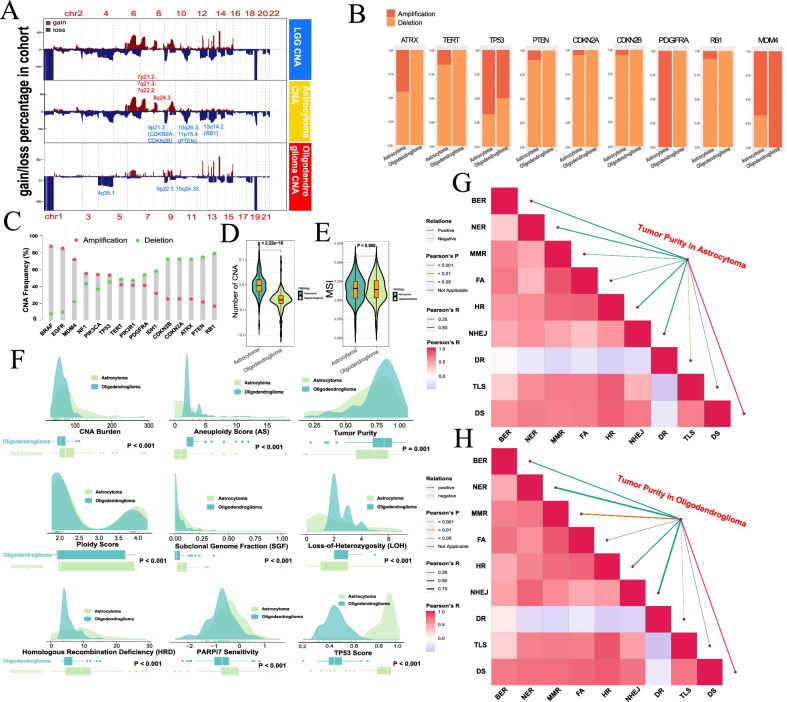
Copy number alterations and DNA damage repair-related signatures. **A** Genomic overview showing the percentage of samples with CNA in astrocytoma, oligodendroglioma and astrocytoma + oligodendroglioma. Some landmark genes and their locations are depicted. **B** Proportion of amplification and deletion of key genes/driver genes between astrocytoma and oligodendroglioma. **C** Rankings of CNA frequency referring to gain and loss status in astrocytoma + oligodendroglioma. **D** Number of CNAs between astrocytoma and oligodendroglioma (Wilcoxon P < 2.22e−16). **E** MSI between astrocytoma and oligodendroglioma (P = 0.900). **F** Quantification of DDR-related signatures in astrocytoma and oligodendroglioma (Wilcoxon rank-sum test). **G** Correlation between DDR-related pathways and tumor purity in astrocytoma. **H** Correlation between DDR-related pathways and tumor purity in oligodendroglioma. Pearson’s correlation analyses were conducted between tumor purity of astrocytoma/oligodendroglioma and each DDR-related pathway, and among the pathways. The coefficients were reported respectively. *CNA* copy number alternation, *AS* aneuploidy score, *DDR* DNA damage repair, *SGF* subclonal genome fraction, *LOH* loss-of-heterozygosity, *HRD* homologous recombination defect, *PARPi* poly (ADP-ribose) polymerase inhibitors, *BER* base excision repair, *NER* nucleotide excision repair, *MMR* mismatch repair, *FA* Fanconi anemia, *HR* homologous recombination, *NHEJ* nonhomologous end joining, *DR* direct repair, *TLS* translesion synthesis, *DS* damage sensor

The distribution of CNA percentage across all chromosomes is shown in Fig. 2A. Astrocytoma demonstrated irregular CNA distribution of focal amplifications (7p21, 7q21, 7q22, 8q24) and deletions (9p21, 10q26, 13q14), and in addition to 1p/19q codeletion, oligodendroglioma demonstrated focal deletions of 4q35, 9p22, 10q24, etc., in which 4q35 had the highest CNA percentage. Astrocytoma exhibited significantly higher amplification proportions of ATRX, TP53 and *PDGFRA*, except *MDM4*, and no amplification status for *ATRX*, *TERT*, *PTEN*, *CDKN2A*/*CDKN2B* or *RB1* was identified in oligodendroglioma (Fig. 2B; Additional file 2: Fig. S2, Additional file 9: Table S2). Astrocytoma exhibited a higher total CNA than oligodendroglioma, but their MSIs were comparable (Fig. 2D, E). In the two kinds of tumors, *BRAF* and *EGFR* exhibited the highest amplification frequency, and *RB1* and *PTEN* displayed the highest deletion frequency (Fig. 2C). Previous studies have shown a close association between CNA and DDR (Tang and Amon 2013; Taylor et al. 2018). Regarding DDR signatures calculated using the ABSOLUTE algorithm, astrocytoma showcased a higher CNA burden, ploidy score, subclonal genome fraction (SGF), homologous recombination deficiency (HRD) score, sensitivity to poly (ADP-ribose) polymerase inhibitors 7 (PARPi7) and TP53 score, while oligodendroglioma exhibited a higher aneuploidy score (AS), tumor purity and loss-of-heterozygosity (LOH) (Fig. 2F; Additional file 9: Table S3). Glioma purity maintains a subtle homeostasis for tumor and stromal cells and is an intrinsic characteristic in developing a suitable microenvironment (Aran et al. 2015; Zhang et al. 2017). Both entities revealed a significant correlation between tumor purity and other DDR-related pathways, except the damage sensor (DS), and tumor purity was negatively associated with direct repair (DR) (Fig. 2G, H; Additional file 9: Table S4). Both entities offered some extent of genomic instability, but the CNA distributions were variable. Although there were similar associations between tumor purity and DDR pathways, there was still considerable variance in DDR signatures.

### Prognostic analyses on tumor subtyping

**Fig. 3:**
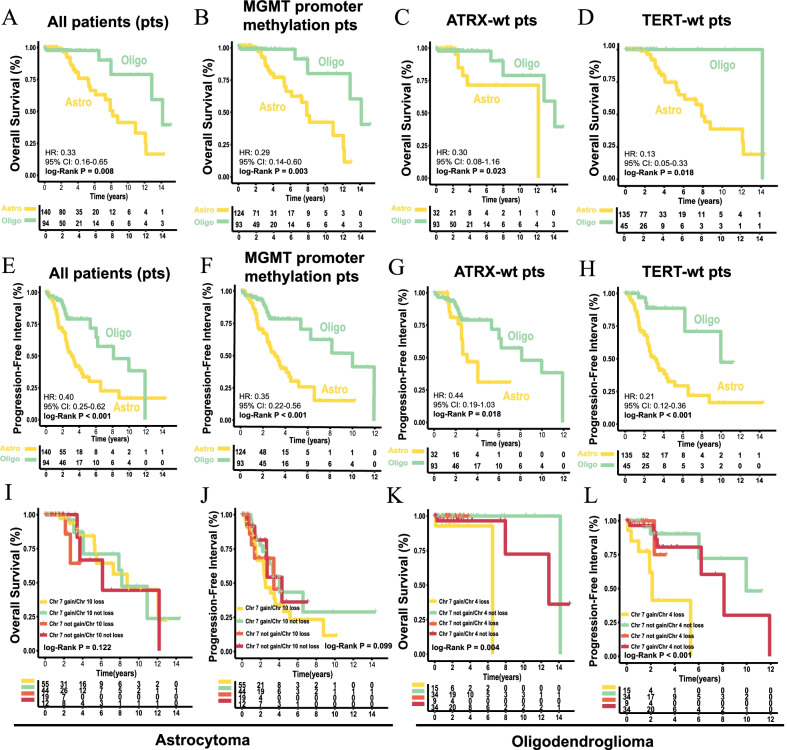
Prognostic analyses based on tumor subtyping. **A–D** Overall survival (OS) of all patients (**A**), MGMT promoter methylation patients (**B**), *ATRX* wild type patients (**C**) and *TERT* wild type patients (**D**) between astrocytoma and oligodendroglioma. **E–H** Progression-free interval (PFI) for all patients (**E**), MGMT promoter methylation patients (**F**), *ATRX* wild type patients (**G**), and *TERT* wild type patients (**H**) between astrocytoma and oligodendroglioma. **I-J** OS (**I**) and PFI (**J**) for astrocytoma patients regarding chr 7/chr 10 status. **K–L** OS (**K**) and PFI (**L**) for oligodendroglioma patients regarding chr 7/chr 4 status. *OS* overall survival, *PFI* progression-free interval, *Astro* astrocytoma, *Oligo* oligodendroglioma, *pts* patients. P-value was calculated by log-rank test

IDH-mt, oligodendroglioma grade 2 exhibited better overall survival (OS) and progression-free interval (PFI) than astrocytoma and in all patients (OS: hazard ratio (HR) 0.33, 95% confidence interval (CI) 0.16–0.65; PFI: HR 0.40, 95% CI 0.25–0.62, Fig. 3A, E) and distinct subgroups of no *O*-6-methylguanine-DNA methyltransferase (MGMT) promoter methylation (OS: HR 0.29, 95% CI 0.14–0.60; PFI: HR 0.35, 95% CI 0.22–0.56, Fig. 3B, F) ATRX wild type (*ATRX*-wt) (OS: HR 0.30, 95% CI 0.08–1.16; PFI: HR 0.44, 95% CI 0.19–1.03, Fig. 3C, G) and TERT wild type patients (*TERT*-wt) (OS: HR 0.13, 95% CI 0.05–333; PFI: HR 0.21, 95% CI 0.12–0.36, Fig. 3D).

In astrocytoma, no significant OS (Fig. 3I, log-rank P = 0.122) or PFI (Fig. 3J, P = 0.099) differences were observed with respect to chr 7 gain/chr 10 loss status; however, chr 7 gain/chr 10 loss astrocytoma presented the worst PFI trend. Oligodendroglioma with chr 7 gain/chr 4 loss was more prone to worse OS (Fig. 3K, P = 0.004) and PFI (Fig. 3L, P < 0.001). These findings should be validated in larger sample sizes. Based on demographic characteristics, the comprehensive results of univariable and multivariable analyses for astrocytoma and oligodendroglioma, respectively, in the TCGA and CGGA cohorts are summarized in Table 1. Somewhat biases were revealed due to limited sample size included in the Cox-regression model, almost no robust prognosis predictors were identified for the two entities.

**Table 1:** Univariable and multivariable analyses based on clinical information for astrocytoma and oligodendroglioma

	Univariable			Multivariable				Univariable			Multivariable		
Astrocytoma Group	HR (95% CI)	P-value	C-index	HR (95% CI)	P-value	C-index	Oligodendroglioma Group	HR (95% CI)	P-value	C-index	HR (95% CI)	P-value	C-index
TCGA (n = 140) (n/%)							TCGA (n = 94) (n/%)						
Age (y)						0.635	Age (y)						0.959
< 50 (reference, 119/85%)	1			1			< 50 (reference, 66/70.2%)	1			1		
50–59 (14/10%)	0.001 (0–Inf)	0.998	0.517	0.001 (0–Inf)	0.998		50–59 (17/18.1%)	2.66 × 10^9^ (0–Inf)	1	0.899	3.37 × 10^9^ (0–Inf)	1	
> 60 (7/5%)	0.85 (0.20–3.63)	0.828		0.72 (0.16–3.26)	0.669		> 60 (11/11.7%)	7.72 × 10^–9^ (0–Inf)	0.999		5.72 × 10^–9^ (0–Inf)	0.999	
Gender							Gender						
Female (reference, 67/47.9%)	1			1			Female (reference, 43/45.7%)	1			1		
Male (73/52.1%)	1.03 (0.48–2.20)	0.943	0.509	1.15 (0.51–2.62)	0.737		Male (51/54.3%)	0.24 (0.03–2.14)	0.203	0.763	0.46 (0.03–7.99)	0.593	
Radiotherapy							Radiotherapy						
No (reference, 69/49.3%)	1			1			No (reference, 68/72.3%)	1			1		
Yes (71/50.7%)	1.20 (0.55–2.66)	0.646	0.519	1.22 (0.49–3.05)	0.67		Yes (27/28.7%)	0.46 (0.04–4.88)	0.521	0.611	0.26 (0.02–3.11)	0.29	
Chemotherapy							Chemotherapy						
No (reference, 87/62.1%)	1			1			No (reference, 60/63.8%)	1			1		
Yes (53/37.9%)	1.20 (0.54–2.71)	0.652	0.587	1.17 (0.47–2.93)	0.733		Yes (34/36.2%)	1.4 (0.23–8.54)	0.713	0.361	1.51 (0.22–10.20)	0.67	
MGMT							MGMT						
Unmethylated (reference, 16/11.4%)	1			1			Unmethylated (reference, 1/1.1%)	1			1		
Methylated (124/88.6%)	0.43 (0.10–1.83)	0.251	0.541	0.38 (0.08–1.71)	0.208		Methylated (93/98.9%)	0.3 × 10^–6^ (0–Inf)	0.999	0.505	0.36 (0–Inf)	1	
ATRX							TERT						
MT (reference, 108/77.1%)	1			1			MT (reference, 49/52.1%)	1			1		
WT (32/22.9%)	0.97 (0.39–2.43)	0.956	0.473	0.88 (0.33–2.31)	0.792		WT (2/2.1%)	0.31 (0.04–2.67)	0.284	0.737	0.52 (0.02–11.03)	0.675	
DNA methylation							Unclear (43/45.7%)	2.34 × 10^–8^ (0-Inf)	0.999		1.3 × 10^–9^ (0–Inf)	1	
G-CIMP-high (reference, 137/97.9%)	1			1									
Codel (3/2.1%)	9.17 × 10^4^ (0–Inf)	0.998	0.506	2.63 × 10^5^ (0–Inf)	0.999		CGGA cohort 1 (n = 80) (n/%)						
							PRS						0.805
CGGA Cohort 1 (n = 100) (n/%)							Primary (reference, 71/88.8%)	1			1		
PRS						0.672	Recurrent (9/11.3%)	20.05 (6.10–65.91)	< 0.001	0.692	19.64 (4.09–94.27)	< 0.001	
Primary (reference, 81/81.0%)	1			1			Age (y)						
Recurrent (19/19%)	1.95 (0.97–3.92)	0.059	0.606	3.08 (1.44–6.59)	0.004		< 50 (reference, 70/87.5%)	1			1		
Age (y)							50–59 (8/10%)	9.59 (1.14–80.40)	0.037	0.599	6.88 (0.53–88.75)	0.139	
< 50 (reference, 88/88%)	1			1			> 60 (2/2.5%)	2.83 (0.79–10.12)	0.11		0.64 (0.12–3.34)	0.56	
50–59 (11/11%)	2.69 (0.36–19.87)	0.334	0.556	1.75 (0.22–13.75)	0.593		Gender						
> 60 (1/1%)	1.93 (0.81–4.62)	0.137		2.4 (0.98–5.89)	0.055		Female (reference, 34/42.5%)	1			1		
Gender							Male (46/57.5%)	0.97 (0.37–2.55)	0.951	0.508	1.09 (0.38–3.13)	0.868	
Female (reference, 37/37%)	1			1			Radiotherapy						
Male (64/64%)	0.59 (0.32–1.09)	0.092	0.538	0.49 (0.26–0.95)	0.033		No (reference, 13/16.3%)	1			1		
Radiotherapy							Yes (67/83.7%)	0.70 (0.16–3.18)	0.647	0.534	0.53 (0.11–2.60)	0.434	
No (reference, 18/18%)	1			1			Chemotherapy						
Yes (82/82%)	1.86 (0.73–4.75)	0.194	0.551	2.18 (0.81–5.85)	0.122		No (reference, 50/62.5%)	1			1		
Chemotherapy							Yes (30/37.5%)	3.46 (1.29–9.29)	0.014	0.605	2.2 (0.74–6.53)	0.155	
No (reference, 54/54%)	1												
Yes (46/46%)	1.52 (0.82–2.81)	0.183	0.545	1	0.403								

*HR* hazard ratio, *95% CI* 95% confidence interval, *MGMT* O6-methylguanine-DNA methyltransferase, *ATRX* alpha thalassemia/mental retardation, X-linked, *TERT* telomerase reverse transcriptase, *MT* mutant, *WT* wild type, *inf* inferential statistics (extremum in statistics)

### Multiomics data regarding the two entities

**Fig. 4:**
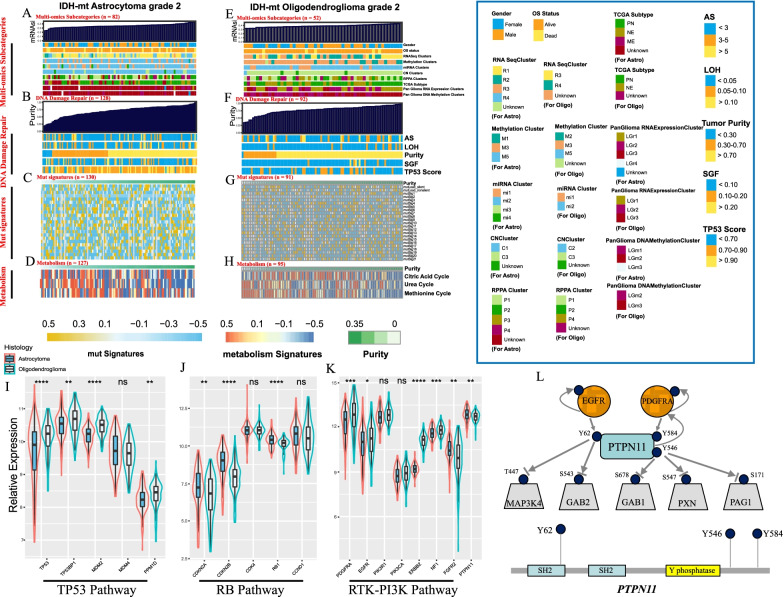
Landscape of multiomics characteristics and DDR signatures in association with the two entities. **A, E** Clinical and multiomics subcategories in astrocytoma (**A**) and oligodendroglioma (**E**) with increased mRNAsi. **B**, **F** DDR signatures in astrocytoma (**B**) and oligodendroglioma (**F**) with increased tumor purity (Astrocytoma vs. oligodendroglioma in SGF: Wilcoxon P < 0.001, in TP53: Wilcoxon P < 0.001). **C, G** Mutation signatures of DDR in astrocytoma (**C**) and oligodendroglioma (**G**) with increased tumor purity. **D**, **H** Metabolic activities in astrocytoma (**D**) and oligodendroglioma (**H**) with increased tumor purity. **I** Relative expression of genes in the *TP53* pathway in tumors. **J**. Relative expression of genes in the *RB* pathway in tumors. **K** Relative expression of genes in the *RTK-PI3K* pathway in tumors (Wilcoxon test, *P < 0.05; **P < 0.01; ***P < 0.001; ****P < 0.0001; ns, not significant). **L** Schematic showing dual regulation of *PTPN11* by *EGFR* and *PDGFRA* and their downstream substrates. *mRNAsi* mRNA-expression-based stemness classifiers

According to the multiomics subcategories (Additional file 9: Table S5), most astrocytomas were classified into methylation cluster M5 (72.0%), CN cluster C1 (87.8%) and panglioma DNA methylation cluster LGm2 (86.6%), while most oligodendrogliomas were assigned to M3 (75.0%), C3 (92.3%), and LGm3 (57.7%). Most samples of astrocytoma (72.0%) and oligodendroglioma (84.6%) were of miRNA cluster mi2 (Fig. 4A, E). Astrocytoma had higher SGF and TP53 scores than oligodendroglioma; however, there were no significant changes in DDR or DDR mut signatures with increased tumor purity (Fig. 4B, C, F, G). Heatmaps further revealed that DNA methylation and RPPA levels were positively associated with tumor purity in oligodendroglioma (Additional file 3: Fig. S3D, F), and a negative association was detected between purity and miRNA level (Additional file 3: Fig. S3E) (Additional file 9: Table S6). No such association was observed in astrocytoma (Additional file 3: Fig. S3A–C). Interestingly, we noticed that with increased tumor purity, metabolic activities of the citric acid cycle (Spearman R = − 0.36, P < 0.001 for astrocytoma; R = − 0.20, P = 0.024 for oligodendroglioma), urea cycle (R = − 0.62, P < 0.001; R = − 0.4, P < 0.001) and methionine cycle (R = − 0.22, P = 0.038; R = − 0.11, P = 0.210, respectively) were inhibited (Fig. 4D, H) potentially due to reduced intratumor heterogeneity in high purity tumors (Wang et al. 2021). The relative expression levels of biomarkers in the TP53 pathway (Fig. 4I) and RTK-PI3K pathway (Fig. 4K) were mostly higher in oligodendroglioma, except for *FGFR2* and *PTPN11*. Astrocytoma exhibited increased or increased signals of expression of RB pathway biomarkers (Fig. 4J). Both *EGFR* and *PDGFRA* regulated the phosphorylation of *PTPN11*, and another study indicated that *PTPN11* mutation was enriched in tumors responsive to anti-programmed death 1 (PD-1) therapy (Wang et al. 2021; Zhao et al. 2019). The dual regulation by *EGFR* and *PDGFRA* and the downstream substrates that PTPN11 might dephosphorylate are shown in Fig. 4L. *PTPN11* might act as a signaling hub for multiple RTKs.

### Immune cell infiltration and immune therapy response

**Fig. 5:**
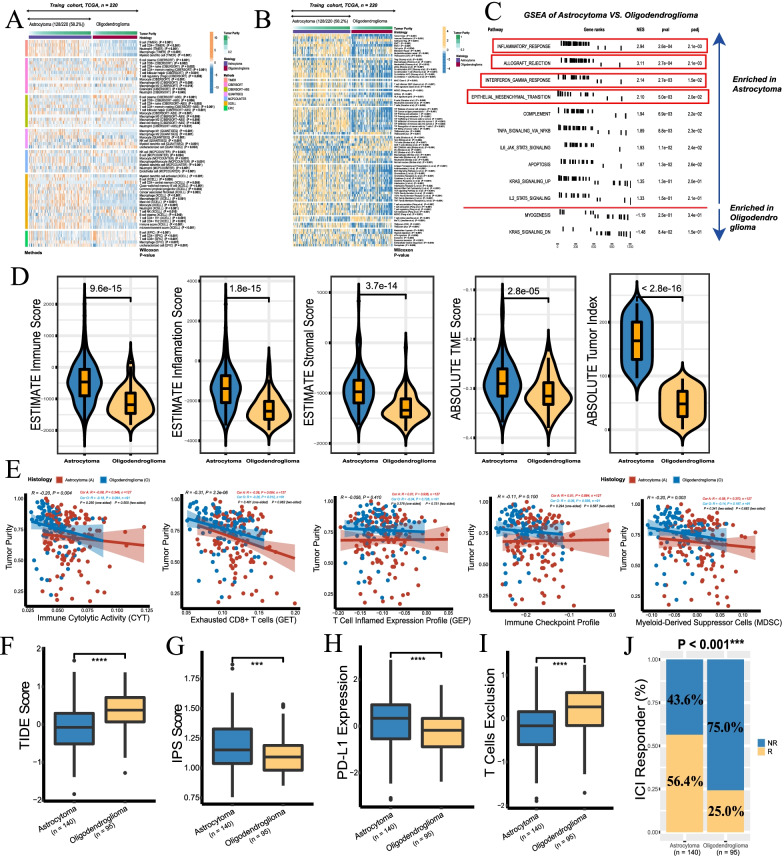
Immune infiltration landscape and checkpoint immunotherapy response in tumors. **A** Immune cell infiltration calculated using multiple computational methods in tumors. Wilcoxon test P-value was attached behind the cell name. **B** Multiple immune and TME signatures calculated using the IOBR method in tumors. Wilcoxon P-value was attached behind the cell name. **C** GSEA indicates an enhanced immune phenotype enriched in astrocytoma. **D** TME and tumor-related scores quantified using ESTIMATE/ABSOLUTE algorithms (Wilcoxon rank-sum test). High immune score and inflammation score indicates infiltrative immune cells, high stromal score indicated large proportion of stromal cells, higher TME score indicates large proportion of nontumor cells (primarily immune + stromal cells), high tumor index indicates great tumor malignancy and progression. **E** Correlation between tumor purity and immune cell function. Spearman R with p-values presented. A, astrocytoma, O, oligodendroglioma. **F** Astrocytoma had a lower TIDE score than oligodendroglioma. **G** Astrocytoma had a higher IPS score than oligodendroglioma. **H** Astrocytoma had higher relative PD-L1 expression than oligodendroglioma. **I** Oligodendroglioma had a higher level of T cell exclusion (***Wilcoxon P < 0.001; ****P < 0.0001). **J** The proportion of immunotherapy responders and nonresponders between astrocytoma and oligodendroglioma, Chi-square test P < 0.001 (***P < 0.001). ICI, immune checkpoint inhibitor

Different algorithms were used to evaluate the immune cell infiltration level to overcome bias caused by using only one method. Here, we noticed that immune cell infiltration was overall higher in astrocytoma than in oligodendroglioma, including M0, M1, and M2 macrophages, while CD8 + and CD4 + T cells were highly enriched in oligodendroglioma (Fig. 5A; Additional file 9: Table S7). With consensus clustering, immune cells computed using CIBERSORT could be classified into four clusters, and close interactions among immune cells were observed in oligodendroglioma (Additional file 4: Fig. S4). Among the TME and immune signatures, astrocytoma indicated overall increased infiltration abundance, which included inflamed immune checkpoints, human leukocyte antigen (HLA) signatures, myeloid-derived suppressor cells (MDSCs), and oligodendroglioma presented inflamed dendritic cells (DCs) and CD4 + and CD8 + T cells, similar to previous findings. In addition, astrocytoma exhibited activated epithelial mesenchymal transformation (EMT) and dMMR, TGF-β and TNF pathway functions (Fig. 5B; Additional file 8: Table S8). Interestingly, it also indicated strong hypoxia and exosome secretion biological features more than in oligodendroglioma (Fig. 5B). Hallmark gene sets enriched in TME activities such as inflammatory response, IFN-γ, and EMT were observed in the astrocytoma group (Fig. 5C). Further Spearman correlations between TME signatures and immune cells were similar in both tumor types (Additional file 5: Fig. S5).

Using dual ESTIMATE and ABSOLUTE strategies, astrocytoma was coupled with a striking immune microenvironment and tumor index (Fig. 5D). In the two entities, tumor purity was negatively associated with CYT (Spearman R = − 0.20, P = 0.004), GET (R =  −  0.31, P < 0.001) and MDSCs (R = − 0.20, P = 0.003), and no significant differences were observed between the two entities in those associations (Fig. 5E). Compared to oligodendroglioma, astrocytoma seemed to be prone to respond to checkpoint immunotherapy considering its higher TIDE and lower IPS scores (Fig. 5F, G) and the objective responder proportion of 56.4% compared to 25.0% in oligodendroglioma (P < 0.001) (Fig. 5J). Although higher CD8 + T cell infiltration was observed, one reason potentially contributing to relatively poor anti-checkpoint immunotherapy of oligodendroglioma is its reduced expression of PD-L1 and other checkpoint molecules (Fig. 5H). Another reason is a higher level of T cell exclusion that facilitates T cell dysfunction and immunotherapy resistance (Joyce and Fearon 2015; Voabil et al. 2021) (Fig. 5I). In fact, no OS (HR 1.20, 95% CI 0.60–2.37) or PFI (HR 1.48, 95% CI 0.95–2.33) benefits of immunotherapy responders were obtained in any patients (astrocytoma + oligodendroglioma), suggesting the poor general efficacy of checkpoint immunotherapy in these two entities (Additional file 6: Fig. S6).

### Tumor purity as a key genomic factor

**Fig. 6:**
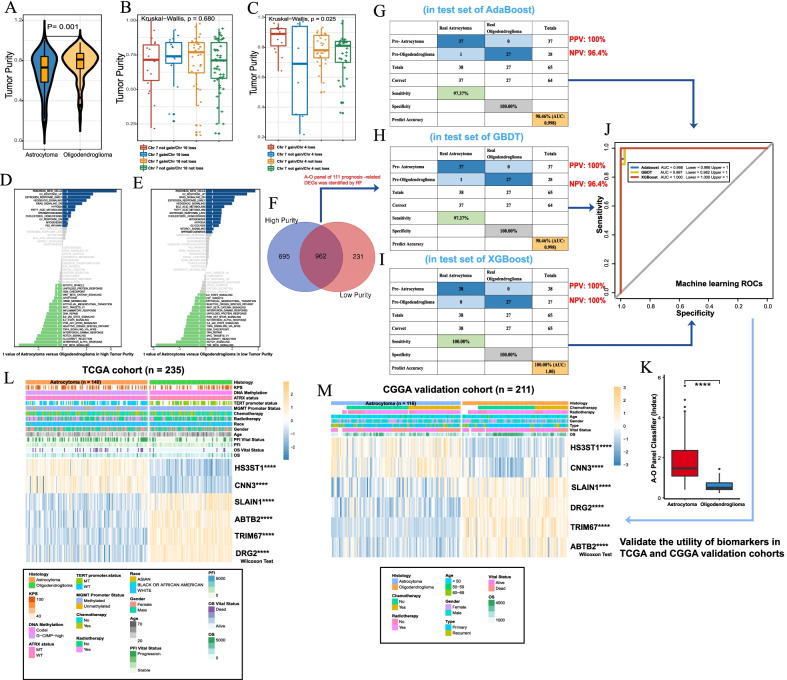
The diagnostic model incorporating the **A**–**O** panel and tumor purity. **A** Oligodendroglioma had higher tumor purity than oligodendroglioma (Wilcoxon P = 0.001). **B** Tumor purity among astrocytoma subtypes based on CNA status (chr 7/chr 10 status, K-W P = 0.690). **C** Tumor purity among oligodendroglioma subtypes based on CNA status (chr 7/chr 4 loss, K-W P = 0.025). **D**, **E** GSVA indicates variation of gene sets enriched in high tumor purity (**D**) and low tumor purity (**E**). Blue bars, gene sets significantly enriched in astrocytoma; green bars, gene sets significantly enriched in oligodendroglioma; gray, not significant. **F**. 962 DEGs selected in high and low tumor purity groups. **G–I** Confusion matrices of binary results of the diagnostic prediction model in the test sets for AdaBoost (**G**), GBDT (**H**) and XGBoost (**I**). **J**. Complex ROCs of three machine learning algorithms. **K** Astrocytoma had significantly higher A–O Panel Classifier (Index) than oligodendroglioma (Wilcoxon ****P < 0.0001). **L** Validation of the 6 important biomarkers of the A–O panel in the TCGA cohort. **M** Validation of the 6 important biomarkers of the A–O panel in the CGGA cohort (Wilcoxon test ****P < 0.0001). *RF* random forest, *ROC* receiver operating curves, *AUC* area under the curve, *PPV* positive predictive value, *NPV* negative predictive value, *OS* overall survival, *PFI* progression-free interval

Glioma purity has been highly associated with major clinical and genomic features in developing a suitable microenvironment (Kioi et al. 2010). We found that oligodendroglioma exhibited higher tumor purity than astrocytoma (Fig. 6A). In view of the CNA status, there were no differences among astrocytomas (Fig. 6B), while oligodendroglioma with chr 7 gain/chr 4 loss exhibited significantly higher tumor purity (Fig. 6C). Using the median point as the cutoff value, samples were assigned to high- and low-purity groups. In both groups, GSVA showed that astrocytoma was associated with hallmark gene sets, such as KRAS signaling, fatty acid metabolism, and hypoxia, and oligodendroglioma was associated with the EMT, DDR, TGF-β, and TNF-α pathways. Then, 962 DEGs between the two groups were identified (Fig. 6F). RF captured 111 prognosis-related DEGs in the A–O panel who with tumor purity was used to establish the diagnostic model (Additional file 7: Fig. S7).

In the test set, AdaBoost (Additional file 8: Fig. S8A, B), GBDT (Additional file 8: Fig. S8C, D), and XGBoost (Additional file 8: Fig. S8E, F) exhibited predictive accuracy for distinguishing astrocytoma from oligodendroglioma of 98.5%, 98.5%, and 100% and AUCs of 0.998, 0.998 and 1, respectively, among which XGBoost demonstrated the best accuracy with an AUC close to the “gold standard” (Fig. 6G–I; Additional file 9: Table S9). ROCs are summarized together in Fig. 6J. All three algorithms displayed consistently excellent performance, demonstrating little overfitting. Formula A–O Panel Classifier used here was: 0.1103 × (expression of *HS3ST1*) + 0.5733 × (expression of *CNN3*) − 0.3355 × (expression of *ABTB2*) + 0.6299 × (expression of *DRG2*) − 0.0292 × (expression of *TRIM67*) − 0.2062 × (expression of *SLAIN1*) (Table S10). In current study, A–O Panel Classifier ranged from 0.2298 to 5.5797 whose median point was 0.9767, and actual astrocytoma ranged from 0.4075 to 5.5797, actual oligodendroglioma ranged from 0.2298 to 1.4337. These findings indicated currently, index less than 0.9767 was more prone to be diagnosed as oligodendroglioma, and higher 0.9767 was to be regarded as astrocytoma (Fig. 6K). Machine learning models yielded excellent identification performance, providing a new diagnostic tool and time window for reasonable intervention. Validation of 6 biomarkers with the highest importance score in TCGA and CGGA cohorts revealed that *HS3ST1*, and *CNN3* were overexpressed in astrocytoma, while *SLAIN1*, *ABTB2*, *TRIM67* and *DRG2* were overexpressed in oligodendroglioma (Fig. 6L, M).

## Discussion

Glioma was one of the earliest tissues exposed to deep genomic and transcriptional analyses, and molecular data and less favorable treatment efficacy both underscore the need for deep insights into the nature of these tumors (Brennan et al. 2013). Current studies have primarily investigated IDH-mt astrocytoma and oligodendroglioma grade 2 but lack integrative analyses. In this study, we primarily conclude the following: (1) oligodendroglioma exhibits a higher percentage of chr 4 loss, and subtypes of chr 7 gain/chr 4 loss indicate poor OS and PFI; (2) the two entities are associated with genomic instability and exhibit marked variation in some DDR signatures; (3) overall, astrocytoma appears to exhibit an infiltrative immune TME and potential response to checkpoint immunotherapy, while oligodendroglioma yielded higher CD4 + , CD8 + T cells as well as T cells exclusion; (4) astrocytoma is more heterogeneous with poor prognosis, while oligodendroglioma seemed to be homogeneous with higher tumor purity and reduced aggression; and (5) machine learning models provide a time window for screening, intervention and clinical decision support. This multidimensional research extends the understanding of diffuse glioma and suggests avenues for further mechanistic analyses of glioma heterogeneity.

Intratumor heterogeneity is a surrogate feature of diffuse gliomas; gliomas with different clonal evolution may exhibit varied characteristics and responsiveness to treatment. Although the stem cell model and stochastic clonal evolution model might explain heterogeneity, they do not indicate the clonal origin of the tumor, and even within the same tumor cell, there was still high clonal heterogeneity, which might confer differential therapeutic sensitivity (Segerman et al. 2016). Glioma heterogeneity analyzed at the mutational, clonal and transcriptional levels suggests a polyclonal evolution of glioma origin rather than a monoclonal origin (Liu et al. 2011). Three or four TCGA subtypes can exist in the same tumor, and single-cell analyses have suggested that the glioma subtype label was similar to the subtype signature of the dominant cell population within the tumor bulk (Patel et al. 2014; Wang et al. 2017). The current study applied multilevel profiling of data to describe inter- and intratumor heterogeneity, tumor purity and TME cell infiltration analyses, potentially identifying dominant cell populations and polyclonal evolution processes.

Both entities suffered genomic instability and exhibited a close relationship to the DDR, and a possible reason for oligodendroglioma exhibiting a higher frequency of chr 4 loss is due to DNA damage during tumor cell evolution. Studies have suggested that the DDR influences carcinogenesis, glioma formation, tumor growth/progression, treatment resistance and multiprofiling of cancer immunogenicity, such as tumor cell-autonomous responses and tumor cell-microenvironment interactions (Carruthers et al. 2018; Chabanon et al. 2021). The standard treatment for glioblastoma (GBM) is associated with inducing DNA damage beyond self-repair. Glioma cells also hijack multiple mechanisms to maintain DNA integrity to circumvent therapeutics, such as single- or double-strand breakage repair, base excision repair (BER), nucleotide excision repair (NER), and mismatch repair (MMR) (Hoeijmakers 2001). Patients with MGMT promoter methylation, which is more common in IDH-mt glioma, exhibited good sensitivity to alkylating agents targeting DNA damage. Furthermore, we found that astrocytoma was more likely to respond to PARP inhibitors (Fig. 2F). Due to their varied origins, somatic mutation status, total CNA number, MGMT methylation proportion (88.6% vs. 98.9%), etc., DDR signatures are distinct between the two entities (Chabanon et al. 2021).

Immunotherapy targeting checkpoints has been approved for a variety of cancers; however, multiple factors influence baseline antitumor immunity. To date, additional therapies targeting T cells, myeloid cells, and other cell types within the complex TME have been promoted, conveying knowledge on the barriers to attaining productive antitumor immunity. For myeloid cell immunosuppression, tumor-associated macrophages (TAMs) often promote angiogenesis, inhibit immune cell function and regulate antitumor immune responses. MDSCs adopt immunosuppressive phenotypes but may induce an antitumor immune response in some situations (Egen et al. 2020). The solid TME also has many factors that promote antitumor immunity, including indoleamine 2,3 dioxygenase 1 (IDO1), TGF-β, VEGFRA, etc. A lack of T cells may indicate a lack of tumor immunogenicity, however, relatively infiltrative CD4/CD8 + T cells in oligodendroglioma do not indicate a considerable checkpoint blockade response (Egen et al. 2020). Similar to GBM, diffuse gliomas have a poor immunotherapy response (Additional file 6: Fig. S6), more importantly, increased T cell exclusion contributes to the refractory TME and circumvents anti-checkpoint immunity (Voabil et al. 2021). Metabolic complexity and flexibility are observed in diffuse gliomas that take up nutrients (glucose, acetate and glutamine, fatty acids and cholesterol) from the extracellular environment and use them for energy and biomass production (Bi et al. 2020). With increased tumor purity, metabolic activities such as the citric acid cycle were increased in these two entities to supply more energy for tumor cells.

In this study, tumor purity was found to be an important genomic factor closely correlated with the DDR and CNA. On the one hand, less proliferative glioma tends to grow slowly and forms a solid bulk with limited nontumor cell infiltration; on the other hand, aggressive gliomas recruit considerable TME cells and use them to create a protective shield (Silver et al. 2016). Accordingly, gliomas with reduced tumor purity are characterized by cellular heterogeneity, aggression and poor prognosis, which indicates that astrocytoma exhibits poor prognosis and marked heterogeneity with low tumor purity. It should be noted that stromal and immune cells are major nontumor fractions, and a tight association between tumor purity and immune function (CYT, GET, MDSCs) has been identified. Different glioma cells selectively recruit immune cells to establish their distinct microenvironment. We found that M2 TAMs and monocytes were enriched in low purity astrocytoma. Although the immunosuppressive TME was created, checkpoint expression was also higher, which catalyzed checkpoint immunotherapy (Gabrilovich and Nagaraj 2009). Unlike single mRNA data analyses, the current study introduced many computational methods and multiomics resources. Innovatively, state-of-the-art machine learning algorithms were leveraged to establish a diagnostic tool with excellent accuracy that identified astrocytomas and oligodendrogliomas. Besides, the tool was efficient, less expensive and minimally invasive because it could give quick histological diagnosis with limited tumor tissue and succinct steps rather than a considerable time after operation for final pathological results. Patients could get timely and correct individualized treatments based on the diagnosis than the previous common treating process. The causality of the biomarkers in the A–O panel has been validated in other studies (Ushakov et al. 2017; Vriend and Tate 2019; Yang et al. 2020). Overall, our study benefits from multiomics data and provides new insights into the clinical, genomic, epigenetic and biological conditions of IDH-mt astrocytoma and grade 2 oligodendroglioma. This diagnostic tool offers support for clinical management.

## Conclusion

This multidimensional investigation adds new insights to the understanding of similarities and differences between IDH-mt astrocytoma and grade 2 oligodendroglioma regarding molecular features, immune microenvironment, tumor purity, classification and prognosis. Rapid advancement in computational algorithms as well as multiomics data will facilitate deeper understanding of diffuse glioma heterogeneity and TME interactions. These findings are ultimately meant to improve patients’ clinical benefits.

## Supplementary Information

**Additional file 1: Fig. S1**. The flow chart of the current study

**Additional file 2: Fig. S2**. Proportion of amplification, deletion, and neutralization of key genes between astrocytoma and oligodendroglioma

**Additional file 3: Fig. S3**. Heatmaps of DNA methylation, miRNA, and RPPA in tumors with increased tumor purity. **A**, **D**. Heatmaps of DNA methylation in astrocytoma (**A**) and oligodendroglioma (**D**). **B**, **E**. Heatmaps of miRNAs in astrocytoma (**B**) and oligodendroglioma (**E**). **C**, **F**. Heatmaps of RPPA in astrocytoma (**C**) and oligodendroglioma (**F**).

**Additional file 4: Fig. S4**. The interactions among 22 immune cells in astrocytoma (**A**) and oligodendroglioma (**B**). The circle size represents the effect of each immune cell type on the prognosis, and the ranges of values calculated using the Cox test were p < 0.5, p < 0.05, p < 0.01, and p < 0.001. Lines linking each immune cell type show the interaction, and the thickness of each line shows the correlation strength. Positive correlations are shown in red, and negative correlations are shown in blue. The infiltration immune cell clusters A-D are marked in yellow, blue, red and brown, respectively.

**Additional file 5: Fig. S5**. The comprehensive Spearman correlation between immune cells and TME-related signatures derived from IOBR. **A**. Distinct correlation in astrocytoma. **B**. Distinct correlation in oligodendroglioma. Cells in blue represent a negative correlation, and cells in red represent a positive correlation. A deeper color indicates a stronger correlation (*Spearman p < 0.05, **p < 0.01, ***p < 0.001).

**Additional file 6: Fig. S6**. Prognostic analyses of checkpoint immunotherapy responders and nonresponders in astrocytoma + oligodendroglioma patients (total n = 234). **A**. Overall survival analyses on distinct patients. **B**. Progression-free interval analyses on distinct patients.

**Additional file 7: Fig. S7**. Selection of prognosis-related A–O panel by RF. **A**. Changes in error rate with number of trees. **B**. Rankings of biomarkers in the A–O panel by variable importance. Biomarkers with higher absolute value of variable importance indicate higher ranking priorities. Blue, protective factors; red, risk factors for overall survival. RF, random forest.

**Additional file 8: Fig. S8**. Detailed results of each machine learning algorithm in establishing the diagnostic model. **A**. Bar plot showing the relative rankings of the A–O panel in Adaboost by discriminative power. **B**. ROC of Adaboost. **C**. Bar plot showing the relative rankings of the A–O panel in GBDT by relative influence. **D**. ROC of GBDT. **E**. Bar plot showing the relative rankings of the A–O panel in XGBoost by importance weight. **F**. ROC of XGBoost. ROC, receiver operating curve. AUC, area under the curve.

**Additional file 9: Table S1**. Data sources summary. **Table S2**. Copy number alternations of key glioma-related genes in the TCGA dataset. **Table S3**. Relative abundance of DNA damage repair signatures in the TCGA dataset. **Table S4**. Relative abundance of DNA damage repair related pathways in the TCGA dataset. **Table S5**. Comprehensive subcategories of intended IDH-mt astrocytoma and oligodendroglioma grade 2. **Table S6**. Differently expressed DNA methylation, microRNA and PRRP between IDH-mt astrocytoma and oligodendroglioma grade 2 in the TCGA dataset. **Table S7**. Wilcoxon test results on immune cells between astrocytoma and oligodendroglioma. **Table S8**. Wilcoxon test results on immune signatures between astrocytoma and oligodendroglioma. **Table S9**. The three machine learning models in identifying astrocytoma from oligodendroglioma. **Table S10**. Multivariable cox regression results for A–O Panel Classifier.

## Data Availability

The datasets used and/or analyzed during the current study are available from the corresponding author on reasonable request.
