# Supplementary figures and images for: Molecular landscape of IDH-mutant astrocytoma and oligodendroglioma grade 2 indicate tumor purity as an underlying genomic factor
Source: Mol Med. 2022 Mar 14;28:34. doi: 10.1186/s10020-022-00454-z (PMC8919570; doi:10.1186/s10020-022-00454-z)

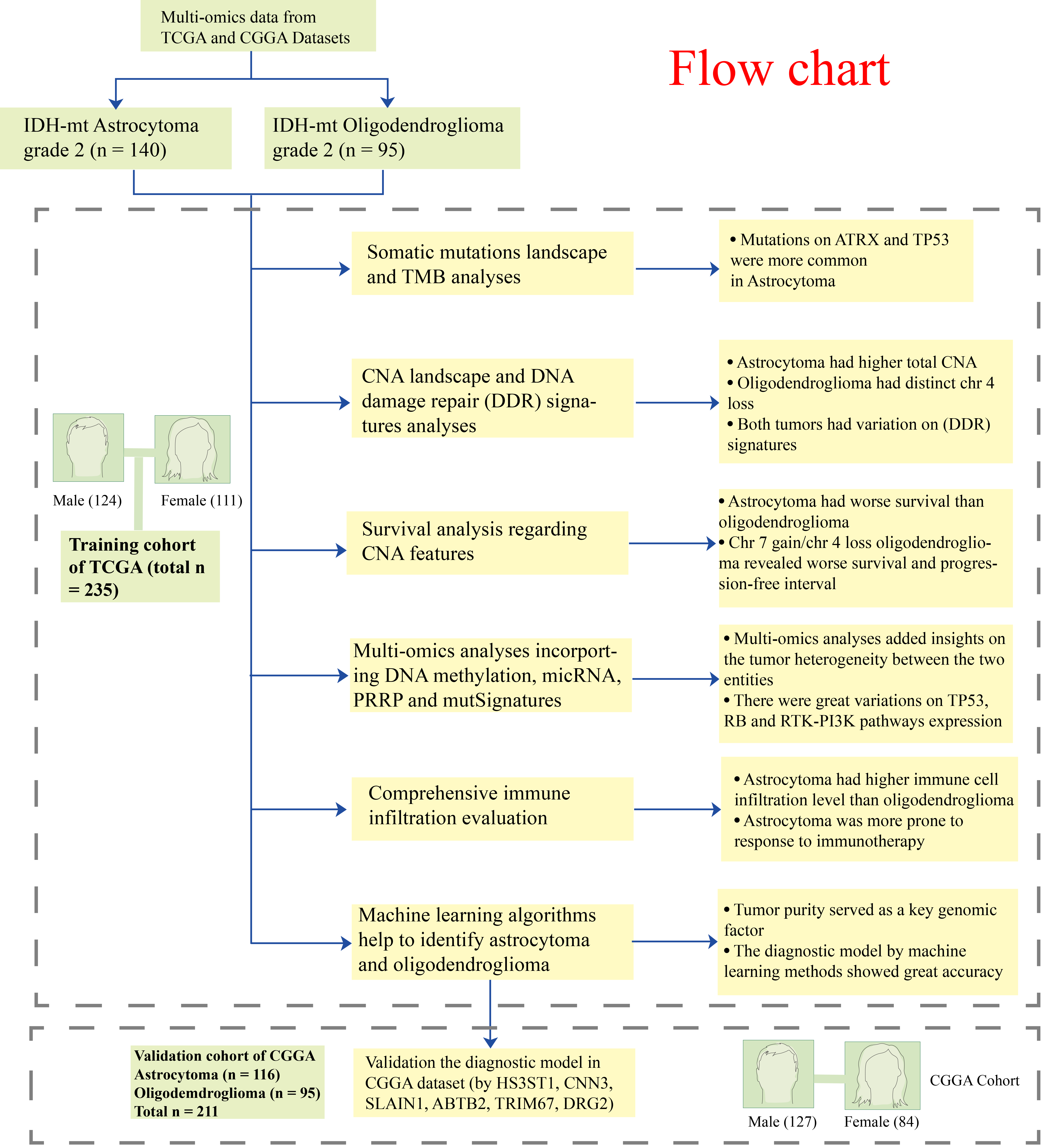

Supplement: Supplementary file 1 — Additional file 1: Fig. S1. The flow chart of the current study [file 10020_2022_454_MOESM1_ESM.tif]

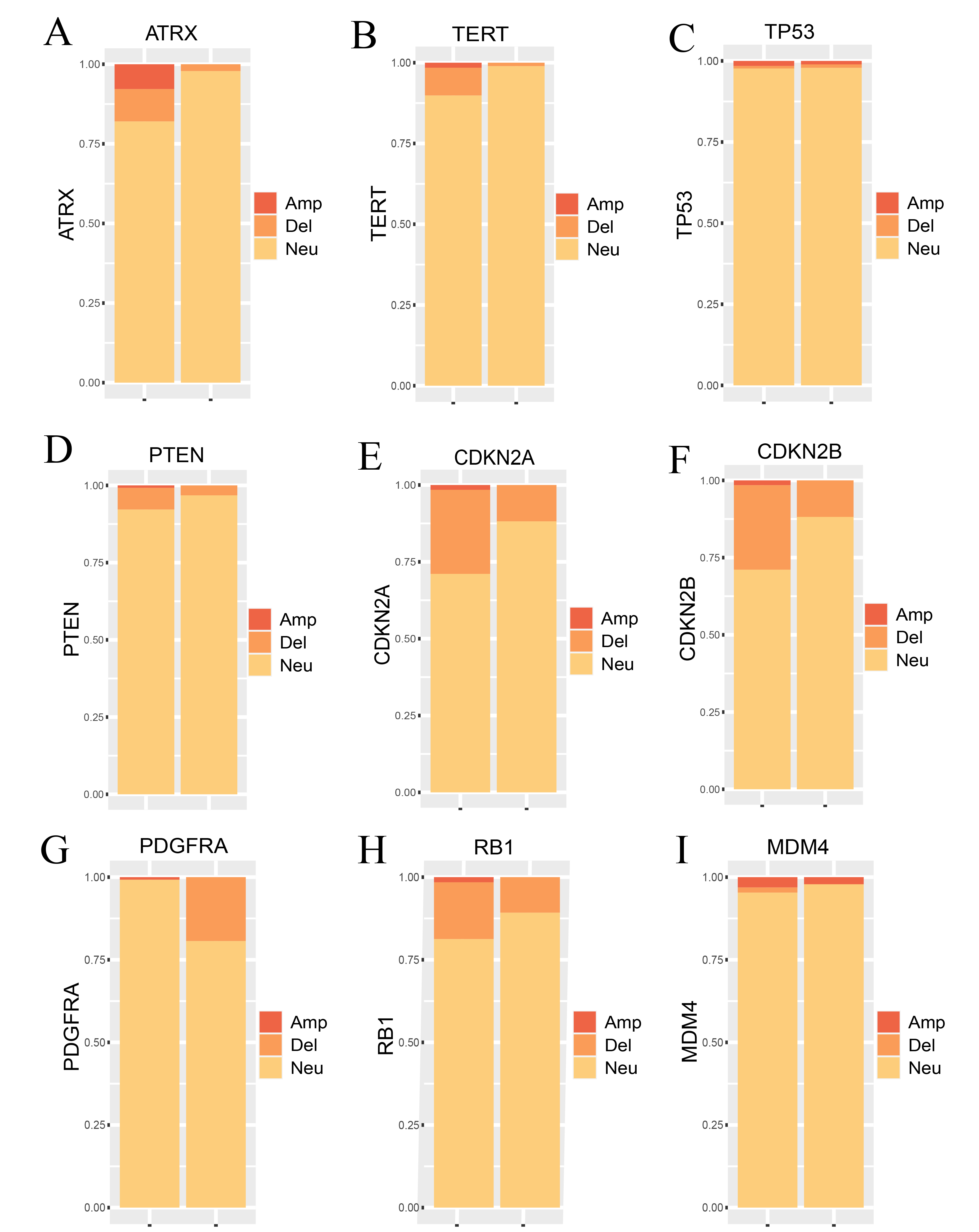

Supplement: Supplementary file 2 — Additional file 2: Fig. S2. Proportion of amplification, deletion, and neutralization of key genes between astrocytoma and oligodendroglioma [file 10020_2022_454_MOESM2_ESM.tif]

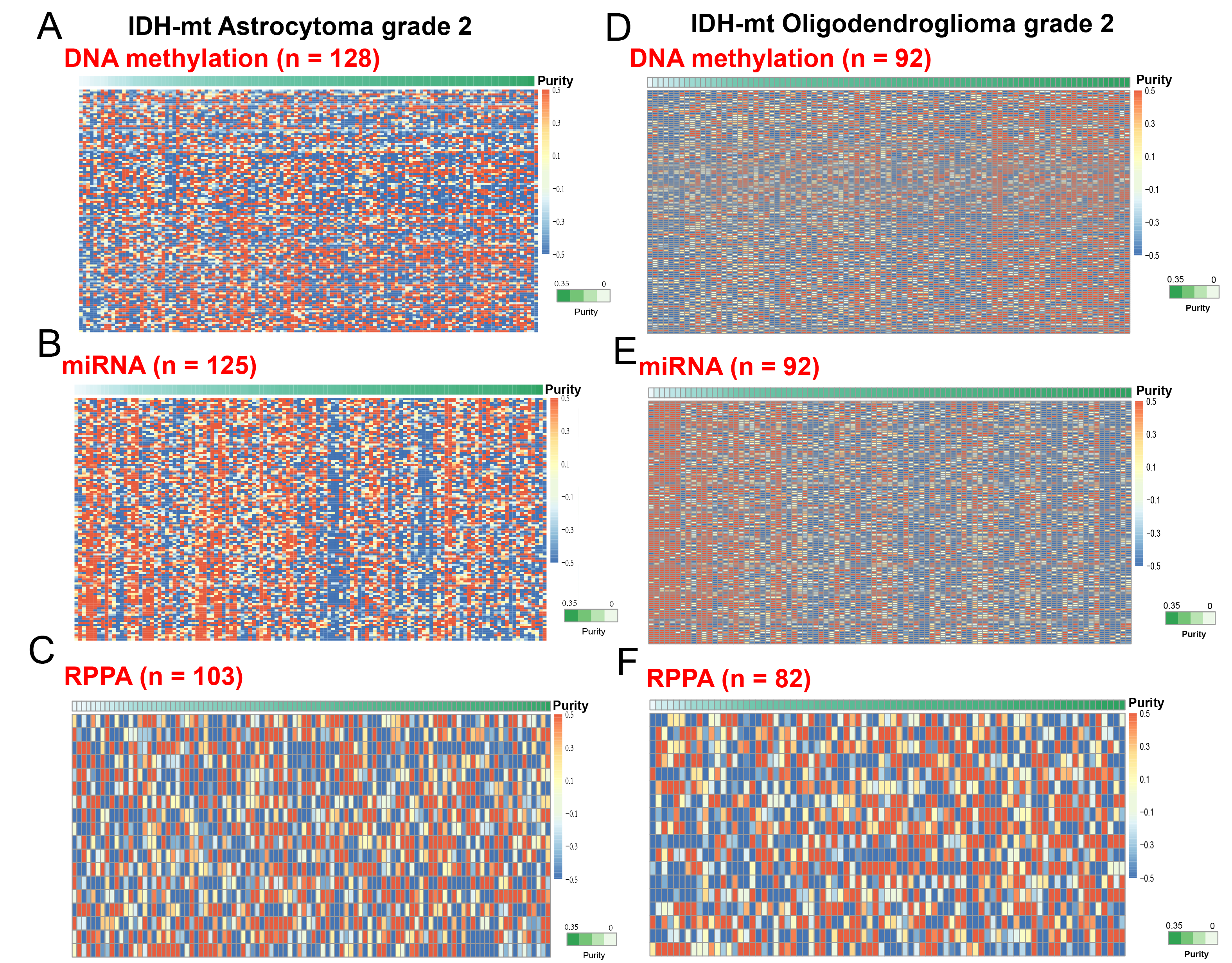

Supplement: Supplementary file 3 — Additional file 3: Fig. S3. Heatmaps of DNA methylation, miRNA, and RPPA in tumors with increased tumor purity. A, D. Heatmaps of DNA methylation in astrocytoma (A) and oligodendroglioma (D). B, E. Heatmaps of miRNAs in astrocytoma (B) and oligodendroglioma (E). C, F. Heatmaps of RPPA in astrocytoma (C) and oligodendroglioma (F). [file 10020_2022_454_MOESM3_ESM.tif]

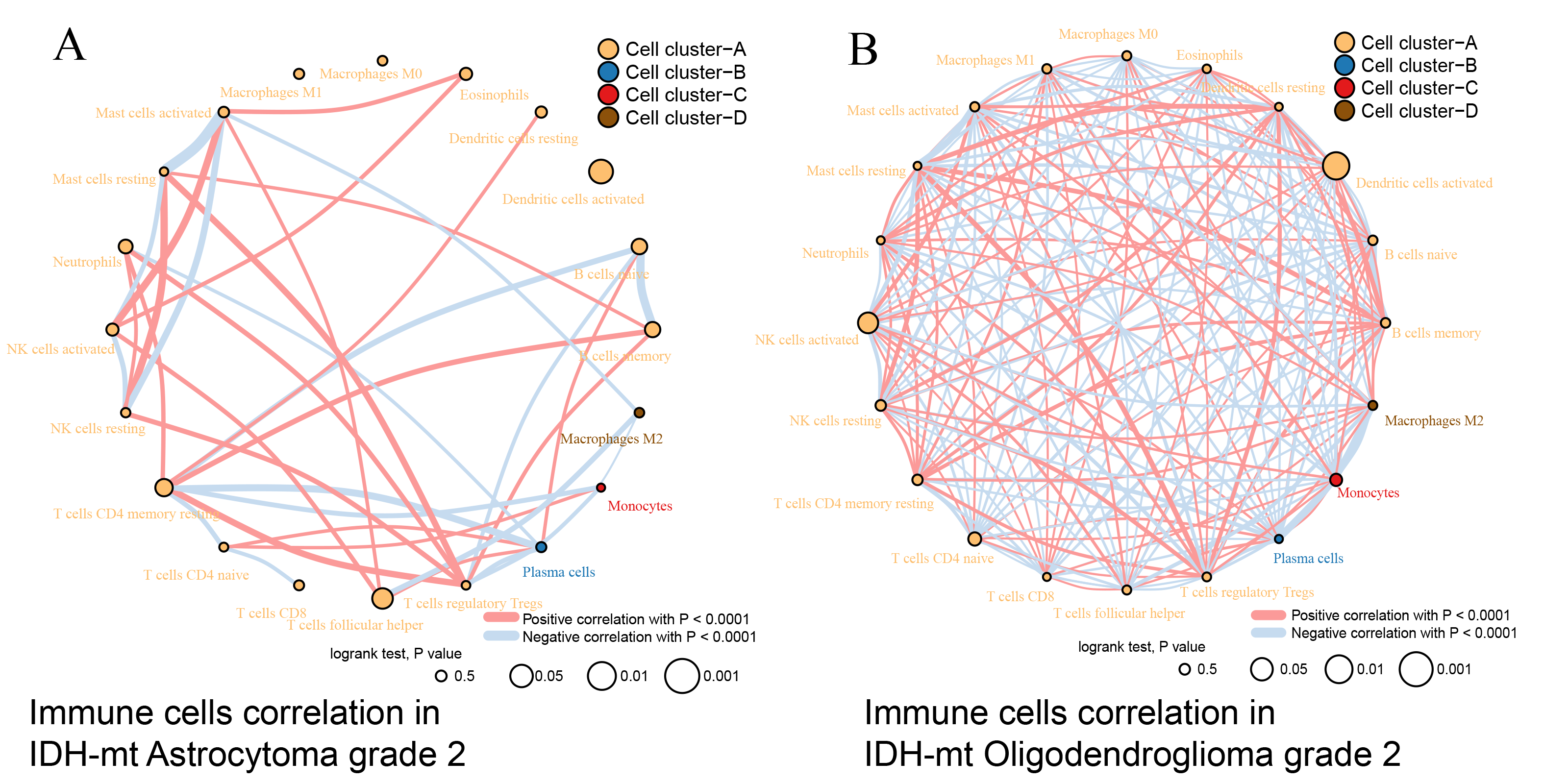

Supplement: Supplementary file 4 — Additional file 4: Fig. S4. The interactions among 22 immune cells in astrocytoma (A) and oligodendroglioma (B). The circle size represents the effect of each immune cell type on the prognosis, and the ranges of values calculated using the Cox test were p < 0.5, p < 0.05, p < 0.01, and p < 0.001. Lines linking each immune cell type show the interaction, and the thickness of each line shows the correlation strength. Positive correlations are shown in red, and negative correlations are shown in blue. The infiltration immune cell clusters A-D are marked in yellow, blue, red and brown, respectively. [file 10020_2022_454_MOESM4_ESM.tif]

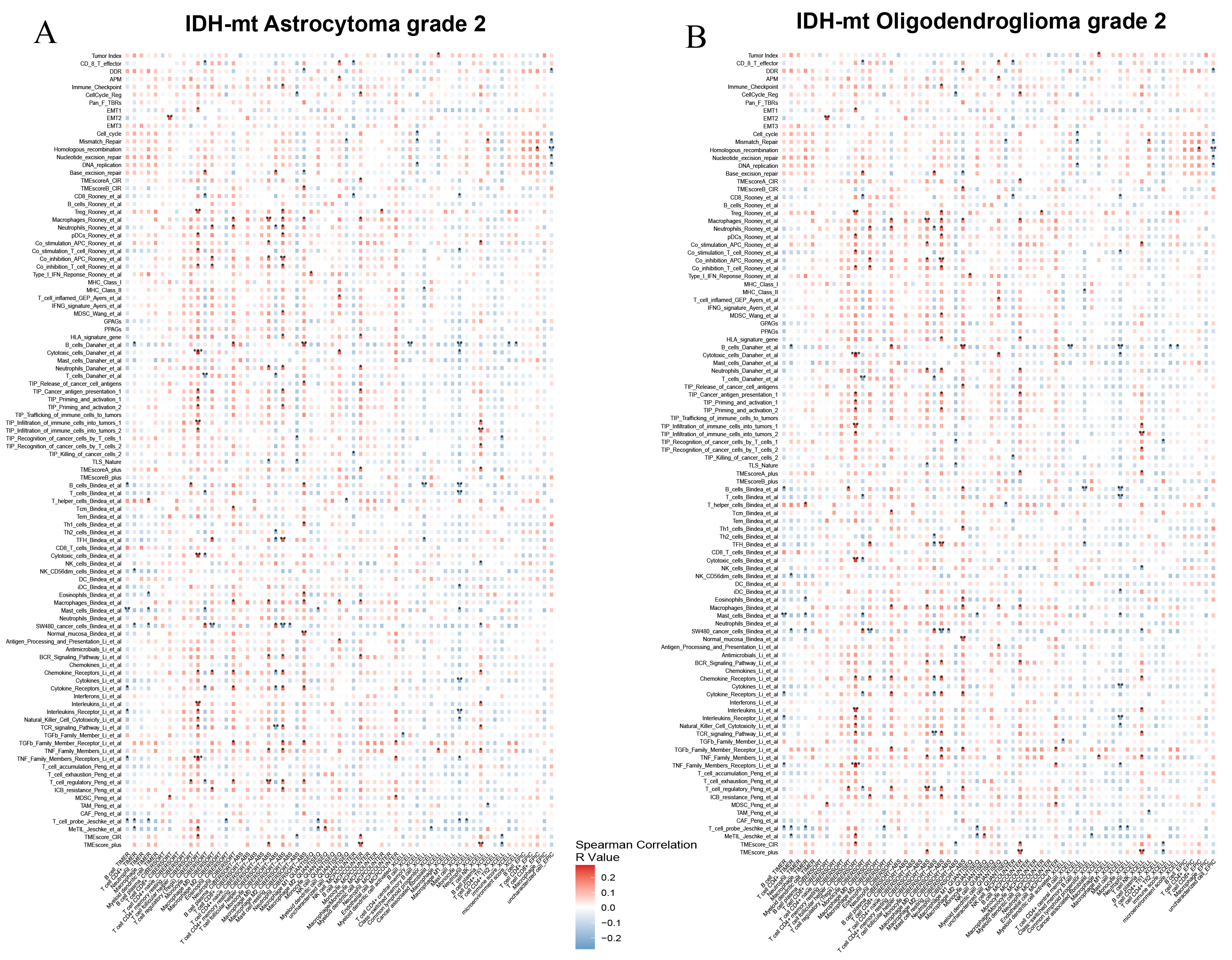

Supplement: Supplementary file 5 — Additional file 5: Fig. S5. The comprehensive Spearman correlation between immune cells and TME-related signatures derived from IOBR. A. Distinct correlation in astrocytoma. B. Distinct correlation in oligodendroglioma. Cells in blue represent a negative correlation, and cells in red represent a positive correlation. A deeper color indicates a stronger correlation (*Spearman p < 0.05, **p < 0.01, ***p < 0.001). [file 10020_2022_454_MOESM5_ESM.tif]

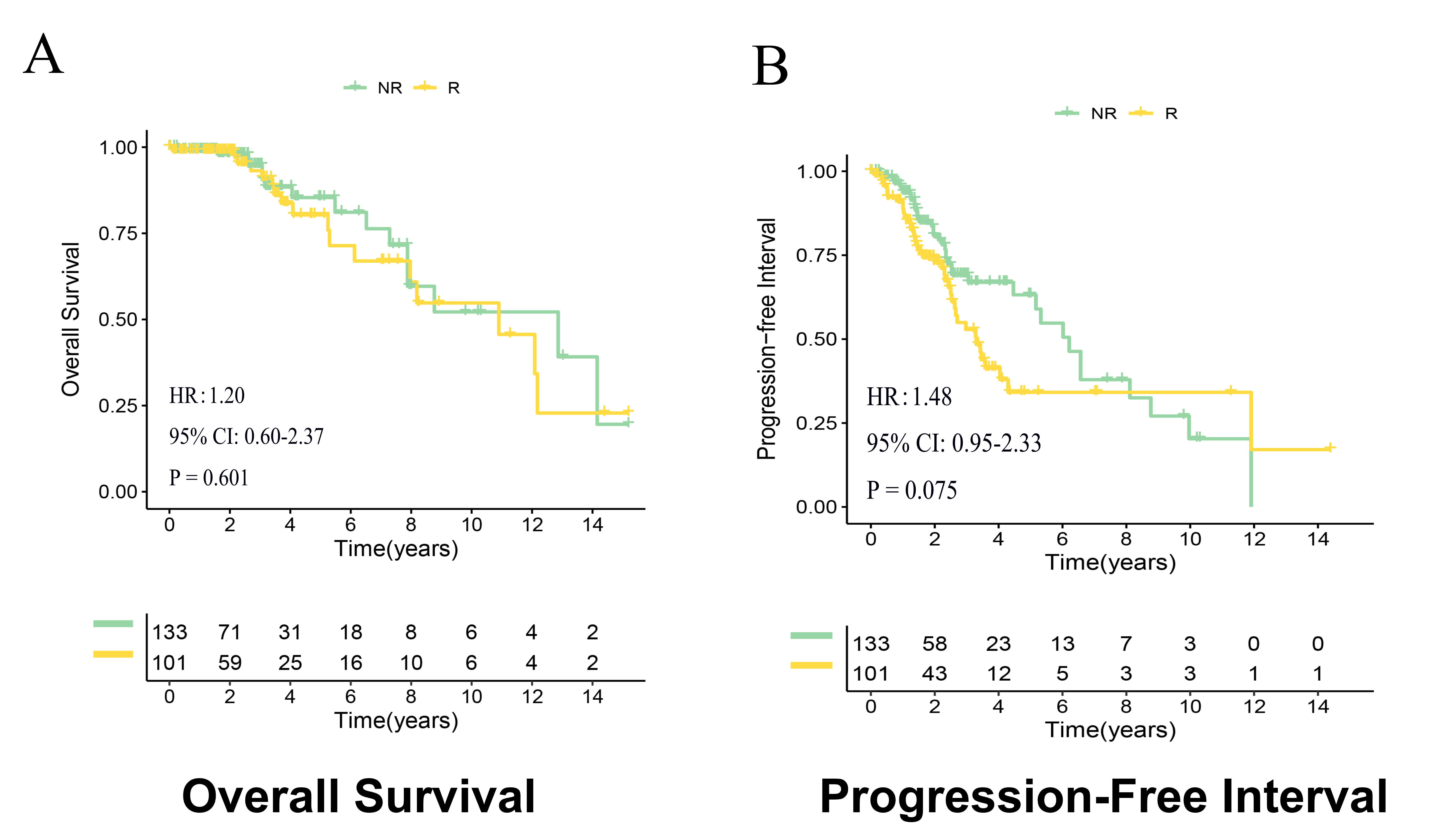

Supplement: Supplementary file 6 — Additional file 6: Fig. S6. Prognostic analyses of checkpoint immunotherapy responders and nonresponders in astrocytoma + oligodendroglioma patients (total n = 234). A. Overall survival analyses on distinct patients. B. Progression-free interval analyses on distinct patients. [file 10020_2022_454_MOESM6_ESM.tif]

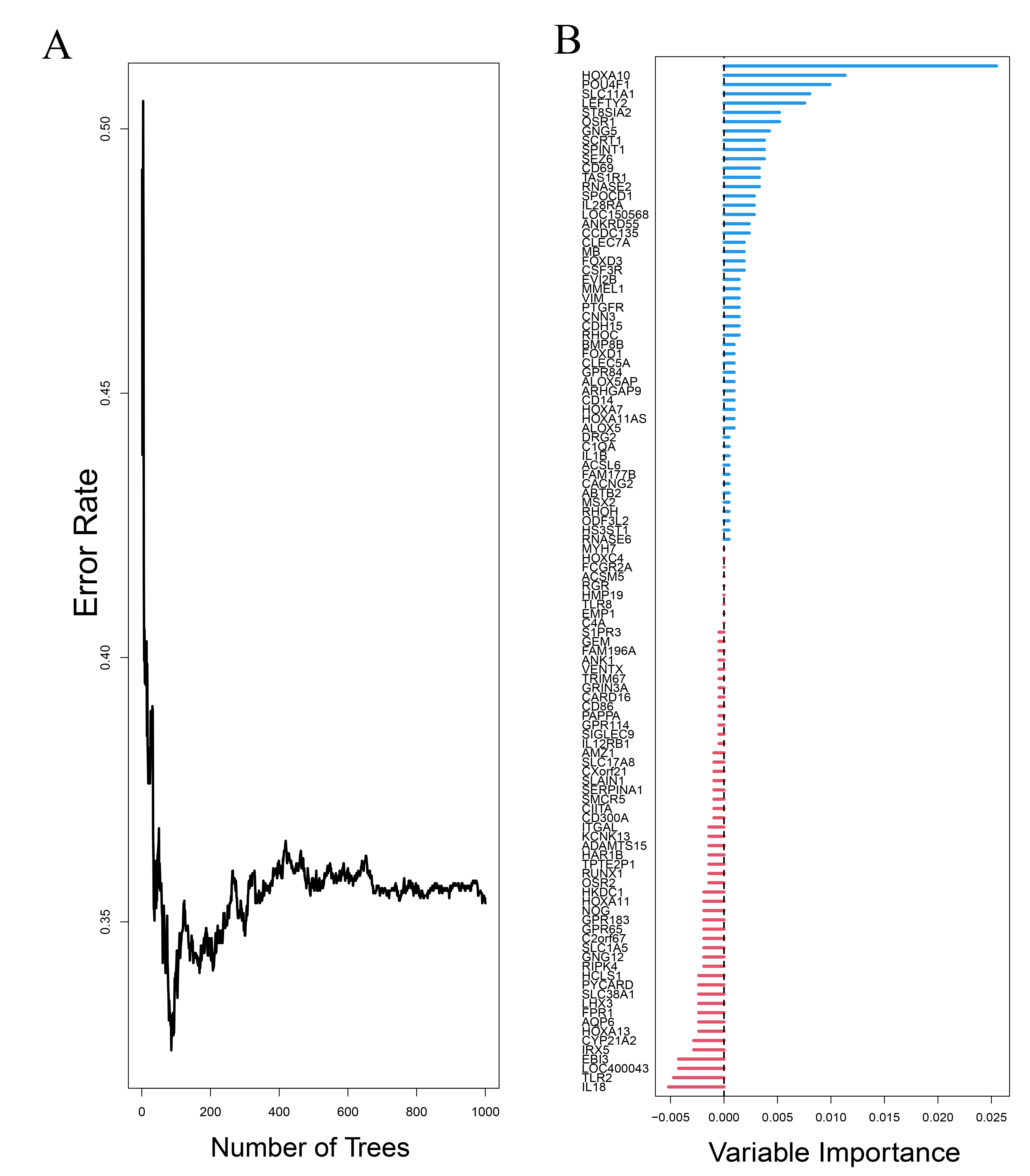

Supplement: Supplementary file 7 — Additional file 7: Fig. S7. Selection of prognosis-related A–O panel by RF. A. Changes in error rate with number of trees. B. Rankings of biomarkers in the A–O panel by variable importance. Biomarkers with higher absolute value of variable importance indicate higher ranking priorities. Blue, protective factors; red, risk factors for overall survival. RF, random forest. [file 10020_2022_454_MOESM7_ESM.tif]

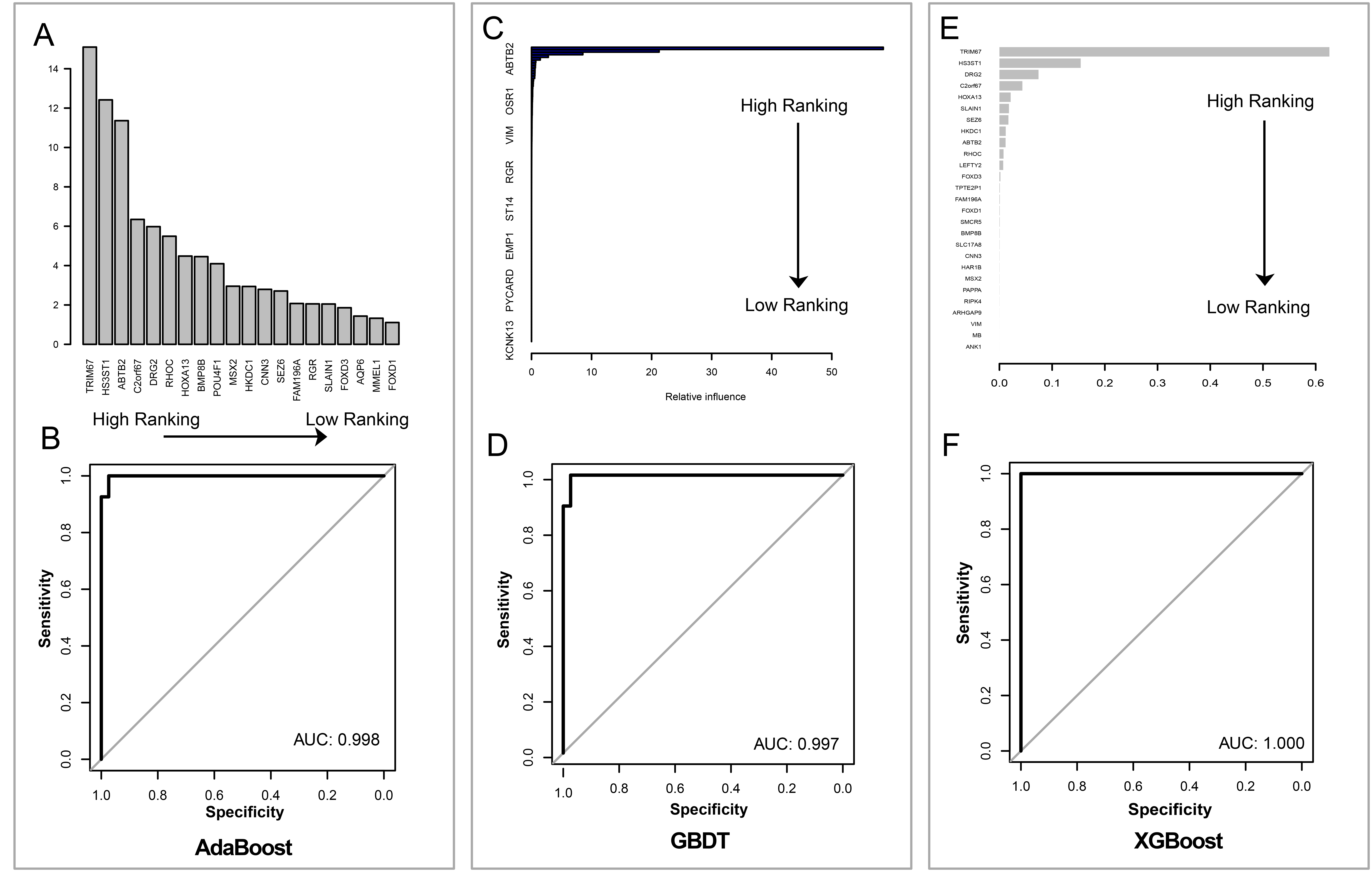

Supplement: Supplementary file 8 — Additional file 8: Fig. S8. Detailed results of each machine learning algorithm in establishing the diagnostic model. A. Bar plot showing the relative rankings of the A–O panel in Adaboost by discriminative power. B. ROC of Adaboost. C. Bar plot showing the relative rankings of the A–O panel in GBDT by relative influence. D. ROC of GBDT. E. Bar plot showing the relative rankings of the A–O panel in XGBoost by importance weight. F. ROC of XGBoost. ROC, receiver operating curve. AUC, area under the curve. [file 10020_2022_454_MOESM8_ESM.tif]
